# Using Renin Activity to Guide Mineralocorticoid Receptor Antagonist Therapy in Patients with Low Renin and Hypertension

**DOI:** 10.1093/ajh/hpad032

**Published:** 2023-04-04

**Authors:** Arian Mansur, Anand Vaidya, Alexander Turchin

**Affiliations:** Division of Endocrinology, Brigham and Women’s Hospital, Boston, Massachusetts, USA; Harvard Medical School, Boston, Massachusetts, USA; Division of Endocrinology, Brigham and Women’s Hospital, Boston, Massachusetts, USA; Harvard Medical School, Boston, Massachusetts, USA; Division of Endocrinology, Brigham and Women’s Hospital, Boston, Massachusetts, USA; Harvard Medical School, Boston, Massachusetts, USA

**Keywords:** blood pressure, hypertension, mineralocorticoid receptor antagonist, proteinuria, primary aldosteronism, renin

## Abstract

**BACKGROUND:**

Mineralocorticoid receptor antagonists (MRAs) are often empirically used for patients with low-renin hypertension (LRH) or probable primary aldosteronism (PA) who decline surgery. However, the optimal approach to MRA therapy is unknown. Studies have shown that a rise in renin is an effective biomarker of prevention of cardiovascular complications of PA. This study aimed to determine whether empiric MRA therapy in patients with LRH or probable PA targeting unsuppressed renin is associated with a decrease in blood pressure and/or proteinuria.

**METHODS:**

Retrospective single-center cohort study from 2005 to 2021 included adults with LRH or probable PA (renin activity <1.0 ng/ml/h and detectable aldosterone levels). All patients were empirically treated with an MRA, targeting renin ≥1.0 ng/ml/h.

**RESULTS:**

Out of 39 patients studied, 32 (82.1%) achieved unsuppressed renin. Systolic and diastolic blood pressure decreased from 148.0 and 81.2 to 125.8 and 71.6 mm Hg, respectively (*P* < 0.001 for both). Similar blood pressure reductions were seen whether patients had high (>10 ng/dl) or low (<10 ng/dl) aldosterone levels. The majority (24/39; 61.5%) of patients had at least one baseline anti-hypertensive medication stopped. Among the six patients who had detectable proteinuria and albumin-to-creatinine (ACR) measurements post-treatment, the mean ACR decreased from 179.0 to 36.1 mg/g (*P* = 0.03). None of the patients studied had to completely stop treatment due to adverse reactions.

**CONCLUSIONS:**

Empiric MRA therapy in patients with LRH or probable PA targeting unsuppressed renin can safely and effectively improve blood pressure control and reduce proteinuria.

## Graphical Abstract

**Graphical Abstract:**
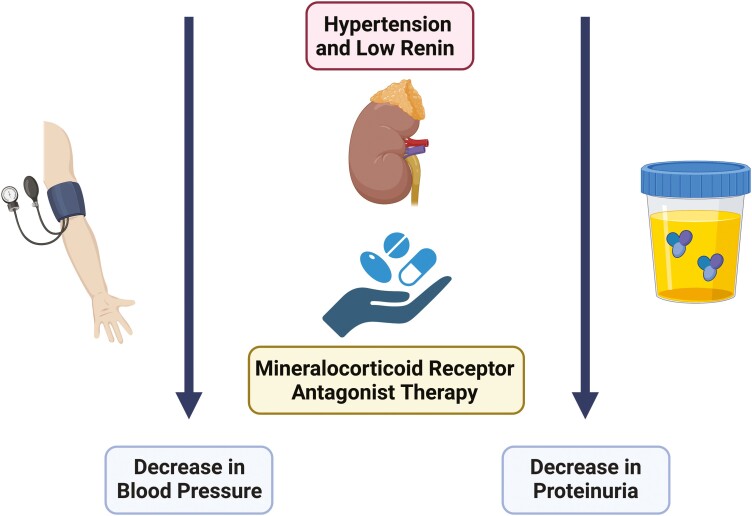

While primary aldosteronism (PA) is often categorized as a discrete condition, recent evidence indicates that PA exists across a broad continuum of pathophysiology, thereby blurring the distinction between PA and low-renin hypertension (LRH).^1–6^ It is widely accepted that mineralocorticoid receptor antagonists (MRAs) can be used for treatment of PA, particularly for patients with non-lateralizing PA, those who are not interested in surgery, or in the settings where surgery may not be available or feasible.^7–9^ However, there are few published studies on the optimal approach of MRA therapy, and there is no consensus about biochemical treatment targets in MRA therapy of PA.^10–14^ Recent literature indicates that MRA therapy in PA is associated with lower risk of cardiovascular disease, but only when treatment results in a substantial rise in renin, from a suppressed to unsuppressed state.^15–21^ However, how to accomplish this in a safe and effective manner is not well understood. We, therefore, conducted a retrospective study that evaluated an approach to treatment of patients with LRH or probable PA with MRAs that involves gradual titration of MRAs targeting unsuppressed renin. We hypothesized that this approach will be associated with a decrease in blood pressure and/or proteinuria in a safe manner.

## METHODS

### Study design

We conducted a retrospective observational cohort study of consecutive patients from the practice of coauthor Alexander Turchin between 1 January 2005 and 31 December 2021. The purpose of this study was to determine whether MRA treatment of patients with LRH or probable PA to induce a rise in renin was associated with a decrease in blood pressure and/or proteinuria.

This study was approved by the institutional review board at Mass General Brigham. The requirement for written informed consent was waived.

### Study population

Study participants included hypertensive patients with suppressed (<1.0 ng/ml/h) plasma renin activity (PRA) and detectable aldosterone levels (≥4 ng/dl) who favored empiric medical therapy over further diagnostic testing for PA (i.e., patients who were not interested in confirmatory testing, adrenal venous sampling, or surgical adrenalectomy). The study participants, therefore, included individuals who probably had PA as well as those who had LRH. All patients were treated with an MRA. MRA therapy was started at 25–50 mg daily of spironolactone (women; chosen based on its low cost) or eplerenone (men; chosen to minimize the risk of gynecomastia and other symptoms of hypogonadism), and the dose was gradually increased in 25–50 mg increments to target a PRA ≥1.0 ng/ml/h or the maximum tolerated dose. Further intensification of the MRA was halted if the patient developed hypotension (asymptomatic systolic blood pressure (SBP) <100 mm Hg or SBP <120 mm Hg associated with lightheadedness), sustained hyperkalemia (K > 5.0 mEq/l) refractory to treatment with diuretics or potassium binders or a sustained decrease (≥25%) in estimated glomerular filtration rate (eGFR).

### Study measurements

Patients’ medical history and demographic information were obtained from the Electronic Medical Record (EMR) system at Mass General Brigham. Patient characteristics that were collected were determined *a priori* and included demographic information (age, sex, and race/ethnicity), baseline measurements (plasma aldosterone levels, PRA, serum potassium, SBP, diastolic blood pressure (DBP), albumin-to-creatinine ratio (ACR), serum creatinine, number of anti-hypertensive medications), pre-existing medical conditions (hypertension, proteinuria (defined as ACR ≥30 mg/g), proteinuria out of proportion to hyperglycemia (defined as ACR ≥30 mg/g and either HbA1c <8.0% (64 mmol/mol) or no diagnosis of diabetes), diabetes, heart failure, and heart failure with reduced ejection fraction), side effects from MRA treatment and use of thiazides, loop diuretics, or potassium binding resins for hyperkalemia. Patients’ blood pressures were measured based on standard methodology utilized in the clinic when the patient was seen, which includes the use of an automatic sphygmomanometer or manually by the auscultation method, with the patient in a seated position. To calculate spironolactone equivalent MRA dose, eplerenone doses were divided by two.^22^ For use in population-level calculations, 0.6 and 0.1 ng/ml/h were used in place of PRA measurements reported as “<0.6 ng/ml/h” and “<0.1 ng/ml/h,” respectively. eGFR was calculated using 2021 version of chronic kidney disease epidemiology collaboration equation.^23^

### Statistical analysis

Summary statistics of patient characteristics were analyzed using measures of central tendency (mean and median) and variation (standard deviation and interquartile range) for continuous variables and frequencies and proportions for categorical variables. Blood pressure, ACR, and other patient characteristics at baseline versus after maximum tolerated MRA dose was achieved were compared using a Wilcoxon signed-rank test. To assess the relationship between the MRA dose needed to achieve unsuppressed renin, a Spearman’s correlation coefficient was calculated, and a linear regression model was constructed with the baseline aldosterone level as a predictor and the spironolactone equivalent MRA dose required to attain unsuppressed renin as the dependent variable.

All statistical analyses were performed using SAS, version 9.4 (SAS Institute, Cary, NC).

## RESULTS

Of the 41 patients with suppressed renin treated with an MRA protocol to target an unsuppressed renin, 39 patients had available information on baseline characteristics and outcomes and were included in the analysis. The median age of study patients was 67 years; all had hypertension; the majority had diabetes (Table 1). Most (30/39; 76.9%) study patients were taking at least three anti-hypertensive medications prior to starting MRA therapy.

**Table 1.** Baseline characteristics of study patients

	All patients (*n* = 39)	Patients who achieved unsuppressed renin (*n* = 32)
Age (years), mean (SD)	66.7 (12.5)	68.0 (11.7)
Sex, No. (%)		
Male	21 (53.8)	17 (53.1)
Female	18 (46.2)	15 (46.9)
Race, No. (%)		
White, non-Hispanic	27 (69.2)	23 (71.9)
White, Hispanic	3 (7.7)	2 (6.3)
Black	7 (18.0)	5 (15.6)
Asian	2 (5.1)	2 (6.3)
Aldosterone (ng/dl), mean (SD)	14.9 (8.8)	14.5 (8.8)
No. of patients with ARR > 20	22 (56.4)	17 (53.1)
No. of patients with ARR > 30	15 (38.5)	12 (37.5)
PRA (ng/ml/h), mean (SD)	0.48 (0.22)	0.49 (0.22)
SBP (mm Hg), mean (SD)	148.0 (18.4)	144.9 (14.6)
DBP (mm Hg), mean (SD)	81.2 (17.5)	78.8 (14.9)
K (mEq/dl), mean (SD)	4.2 (0.5)	4.2 (0.5)
ACR (mg/g) examined		
No. of patients with ACR Examined	18	16
ACR (mg/g), mean (SD)	85.8 (101.7)	94.7 (104.6)
Proteinuria out of proportion to hyperglycemia, No. (%)	7 (38.9)	7 (43.8)
eGFR (ml/min/1.73m^2^) examined		
No. of patients with eGFR Examined	36	30
eGFR (ml/min/1.73m^2^), mean (SD)	71.9 (19.9)	71.6 (15.9)
No. of anti-hypertensive medications, mean (SD)	3.4 (1.4)	3.4 (1.3)
Diabetes, No. (%)	21 (53.9)	19 (59.4)
Heart Failure, No. (%)	7 (18.0)	6 (18.8)
Reduced ejection fraction, No. (%)	1 (2.6)	1 (3.1)
Hypertension, No. (%)	39 (100)	32 (100)

Abbreviations: ARR, aldosterone-to-renin ratio; PRA, plasma renin activity; SBP, systolic blood pressure; DBP, diastolic blood pressure; K, potassium; ACR, urine albumin-to-creatinine ratio; eGFR, estimated glomerular filtration rate; MRA, mineralocorticoid receptor antagonist; IQR, interquartile range; SD, standard deviation.

**Figure 1:**
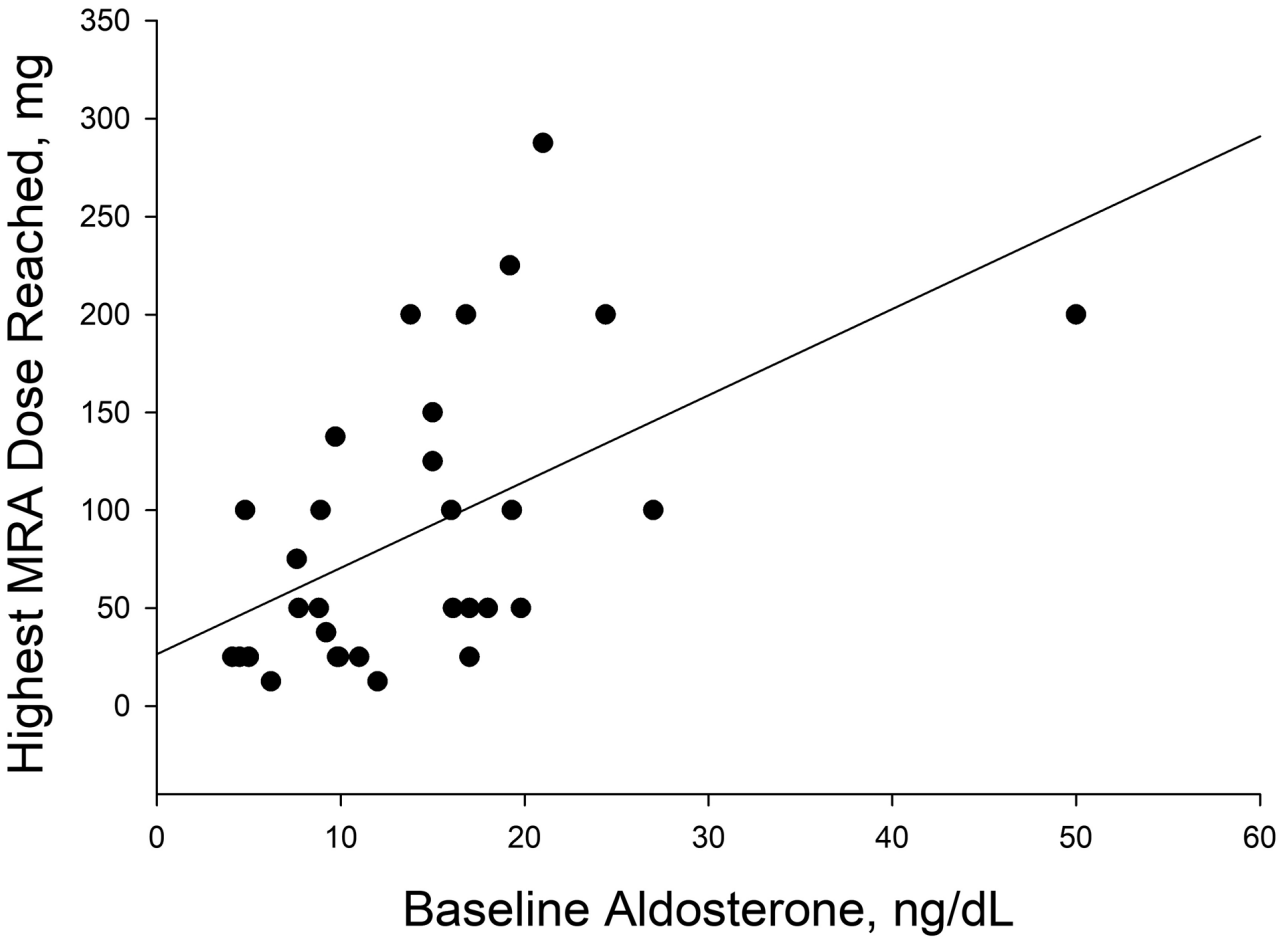
**|** Scatterplot showing relationship between baseline aldosterone levels and highest MRA dose reached in spironolactone equivalent to achieved unsuppressed renin levels. “r” value = 0.52 (moderate correlation). MRA indicates mineralocorticoid receptor antagonist.

MRA therapy was titrated over a median of 59 (IQR 0–322) days involving a mean of 3.6 (SD 3.9) titrations to the median dose of 50 (IQR 25–150) mg of spironolactone equivalent; 12 (30.8%) patients were titrated to a spironolactone equivalent >100 mg daily. Patients who achieved unsuppressed renin were titrated over a median of 54 (IQR to 6.5–344) days involving a median of 2.5 (IQR 1–4) titrations to the median dose of 50 (IQR 25–131.3) mg of spironolactone equivalent. The MRA dose required to achieve unsuppressed renin correlated with the baseline aldosterone levels (Figure 1). On average, the final MRA dose (in mg of spironolactone equivalents) needed to achieve unsuppressed renin was approximately 25 + 5*x, where x is the baseline aldosterone level (in ng/dl).

**Figure 2:**
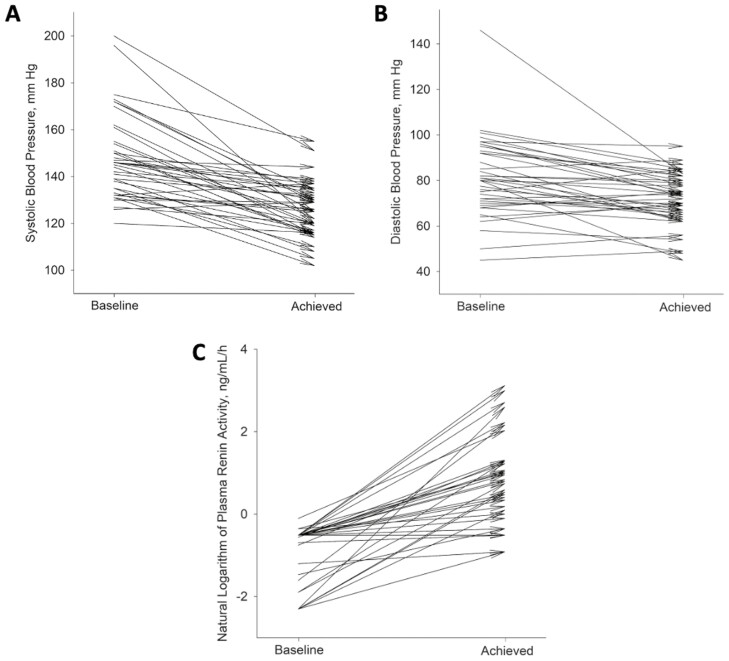
**|** Changes in systolic blood pressure (**a**), diastolic blood pressure (**b**), and plasma renin activity (**c**) at the individual patient level.

Over the course of treatment, mean SBP decreased from 148.0 (SD 18.4) to 125.8 (SD 11.8) mm Hg and mean DBP from 81.2 (SD 17.5) to 71.6 (SD 11.6) mm Hg (*P* < 0.001 for both). Mean PRA increased from 0.49 (SD 0.22) to 4.66 (SD 5.58) ng/ml/h (*P* < 0.001). Changes at the individual patients’ blood pressure and renin levels are shown in Figure 2. Mean aldosterone-to-renin ratio decreased from 55.0 (SD 63.2) to 22.7 (SD 38.0) (*P* < 0.001). The majority (24/39; 61.5%) of patients had at least one baseline anti-hypertensive medication stopped during the course of MRA up-titration. Mean potassium levels increased from 4.18 (SD 0.48) to 4.66 (SD 0.41) mEq/dl (*P* < 0.001). Mean eGFR decreased from 71.9 (SD 19.9) to 61.9 (SD 21.1) ml/min/1.73m^2^ (*P* < 0.001).

In the subgroup of 32 (82.1%) patients who achieved unsuppressed PRA, the mean systolic and DBP decreased from 144.9 (SD 14.6) and 78.8 mm Hg (SD 14.9) at baseline to 125.3 (SD 11.5) and 71.4 mm Hg (SD 11.1), respectively (*P* < 0.001 for both). The mean number of anti-hypertensive medications decreased from 3.4 (SD 1.3) at baseline to 2.4 (SD 1.5) after achievement of unsuppressed PRA (*P* < 0.001).

Among the 15 patients who had baseline aldosterone <10 ng/dl, the mean systolic and DBP decreased from 144.6 (SD 16.5) and 78.4 mm Hg (SD 13.1) at baseline to 121.9 (SD 12.6) and 67.9 mm Hg (SD 9.5), respectively (*P* < 0.01 for both). Patients whose baseline aldosterone was <10 ng/dl had a similar mean decrease in both systolic (22.7 vs. 22.0 mm Hg; *P* = 0.89) and diastolic (10.5 vs. 9.0 mm Hg; *P* = 0.31) blood pressures to the patients whose baseline aldosterone was ≥10 ng/dl. Among the six patients who had detectable proteinuria and ACR measurements post-treatment, the mean ACR decreased from 179.0 (SD 102.1) at baseline to 36.1 (SD 49.2) mg/g (*P* = 0.03).

A total of 15 (38.5%) patients had their MRA dose reduced due to experiencing adverse reactions to MRA treatment, including hyperkalemia, symptomatic hypotension, and acute kidney injury (Table 2). All adverse reactions were successfully treated (e.g., with a decrease in MRA dose). None of the patients had to stop MRA therapy completely due to an adverse reaction.

**Table 2.** Safety of MRA treatment

	All patients (*n* = 39)
Hyperkalemia	7 (17.9)
Hypotension	5 (12.8)
Acute kidney injury	1 (2.6)
Pharmacotherapy for hyperkalemia, No. (%)	2 (5.1)

Abbreviations: MRA, mineralocorticoid receptor antagonist; PRA, plasma renin activity.

## DISCUSSION

In this retrospective observational study, we found that MRA therapy targeting unsuppressed renin levels in patients with LRH or probable PA was associated with substantial decreases in blood pressure and proteinuria, in the context of minimal adverse events that could be easily managed. Importantly, the effectiveness of this treatment approach was similar regardless of whether patients had a likely LRH phenotype (aldosterone <10 ng/dl) or likely PA phenotype (aldosterone >10 ng/dl), implying that empiric MRA therapy in patients with LRH could be an effective and pragmatic approach to achieve blood pressure control and potentially prevent complications in this underdiagnosed and undertreated condition.^24,25^

The treatment strategy of targeting unsuppressed renin was highly effective in lowering blood pressure, with a mean decrease in SBP in excess of 20 mm Hg, despite the majority of patients discontinuing at least one baseline anti-hypertensive medication. This magnitude of blood pressure reduction compares favorably to what has been seen in most studies of other anti-hypertensive medications who had baseline blood pressure at a similar level.^26–28^ Unlike some of the previously reported investigations,^29^ blood pressure reductions were similar between spironolactone and eplerenone, while eplerenone doses were approximately twice as high as spironolactone, consistent with their relative MR antagonist potency.^22^

Another important benefit of MRA treatment in our study was the reduction in proteinuria. The study found a mean decrease in ACR of 79.8% among the patients who had proteinuria. This reduction is greater than that reported in the randomized controlled trials in patients with proteinuria treated by ACEi and ARBs.^30,31^ It is also larger than the 20–30% decrease in ACR reported when patients with proteinuric chronic kidney disease are treated with MRA therapy without regard to baseline or achieved renin levels.^32,33^ Notably, several of the study patients had proteinuria despite having only modest hypertension well controlled with non-MRA anti-hypertensives at study entry. These findings suggest that, in addition to uncontrolled hypertension, unexplained proteinuria in conjunction with suppressed renin could also serve as an indication for MRA therapy.

Treatment with MRAs in our study was safe. No participant had to stop treatment completely due to an adverse reaction. All of the adverse reactions observed in the study, with the most common being hyperkalemia, were reversible. Effective and safe treatments for hyperkalemia, such as sodium zirconium cyclosilicate^34–36^ and patiromer,^37–39^ recently became available and may further facilitate titration of MRA therapy to achieve unsuppressed renin in this patient population.^40^ Even at a relatively high mean spironolactone equivalent dose of nearly 100 mg, none of the men in the study developed gynecomastia or any other symptoms of hypogonadism, as they were all treated with eplerenone.

It is important to note that the MRA dose needed to achieve unsuppressed renin in this series was reached carefully and in gradual 25–50 mg escalations. Based on the results of our study, we encourage clinicians to use our estimated final MRA dose reached of approximately 25 + 5*x, where x is the baseline aldosterone level (in ng/dl), as a guidance instead of a dose that is specifically targeted or started with. It is important to also note that hyperkalemia can happen at any time during dose escalation, even before renin is unsuppressed; therefore, clinicians should carefully monitor patient’s potassium after each escalation.

There is currently no consensus on the optimal approach to or the biochemical treatment targets for MRA therapy in patients with PA. Current Endocrine Society guidelines for medical therapy of PA, published in 2016, recommend MRA titration up to a fixed dose (100 mg of spironolactone), but do not specify targeting a particular renin level.^13^ Other studies have examined MRA treatment focused on the achieved absolute renin levels,^15,16^ the changes in renin levels,^10^ the baseline aldosterone-to-renin ratio,^12^ or the potassium levels.^11^ Importantly, multiple recent studies have shown that the residual cardiovascular risk in PA patients treated with MRA may be limited to individuals with persistently suppressed renin, and that a substantial rise in renin may mitigate this excess risk.^11,15–21^ In the recent report that focused on targeting potassium levels, patients with PA only benefited from intensification of MRA therapy if they had suppressed renin at baseline.^11^ The present study therefore adds to this body of literature that suggests that unsuppressed renin may serve as a biomarker of adequate MR antagonism against activation by excessive aldosterone and patients with suppressed renin and related symptoms (hypertension and/ or proteinuria) should be considered for MRA therapy targeting normal renin levels. As reductions in blood pressure were similar across the spectrum of aldosterone levels and aldosterone-to-renin ratios, it is likely that this approach could be used both for patients with hypertension and suppressed renin and for patients with PA who choose medical therapy.

The present study had a number of strengths. It included an unselected patient population that was treated with a uniform protocol. It offers a pragmatic approach that utilizes tests that are widely available in primary care setting and off-patent medications. Finally, the study found effects that were so considerably pronounced that they were highly statistically significant even in a relatively small population.

The findings presented in this study should be interpreted in light of its limitations. In absence of a control group, the study cannot make any causal claims. The sample size was relatively small. However, because the effects of MRA therapy were nearly uniform, the findings were robust. Because data collected in this analysis came from a single center from the practice of a single physician in eastern Massachusetts, the results may not be generalizable to other settings. Formal testing for PA was not conducted; however, this was deliberate, as the patients opted for pragmatic medical therapy. Other factors that could have affected renin levels (e.g., dietary sodium content or genotype) were not evaluated. The sample size was too small to evaluate for the potential effects of concomitant beta-blocker use. Most patients also could not stop all their non-MRA anti-hypertensive drugs (sometimes because of indications not related to treatment of hypertension), which could influence the explanatory component on blood pressure and renin measures from MRA therapy. We also do not have information on the laboratory methodologic data on the assays used to measure aldosterone and PRA. The sample size was too small to evaluate risk factors for hyperkalemia in study patients. Finally, clinical outcomes were not a focus in our study.

In conclusion, the findings from this study suggest that suppressed renin may be sufficient to identify hypertensive patients who could benefit from MR blockade, irrespective of whether they have LRH or probable PA, and that MRA therapy targeting normal renin levels is effective and safe in this patient population. This study presents a pragmatic approach to the treatment of LRH that could be easily and broadly implemented. Further research is needed to confirm these findings in a controlled study design and to establish long-term clinical effects.

## Data Availability

The de-identified data underlying this article will be shared on reasonable request to the corresponding author, subject to the institutional policy.
